# Attitude of Healthcare Professionals: A Major Limiting Factor in Organ Donation from Brain-Dead Donors

**DOI:** 10.1155/2014/296912

**Published:** 2014-09-30

**Authors:** Maciej Kosieradzki, Anna Jakubowska-Winecka, Michal Feliksiak, Ilona Kawalec, Ewa Zawilinska, Roman Danielewicz, Jaroslaw Czerwinski, Piotr Malkowski, Wojciech Rowiński

**Affiliations:** ^1^Department of General and Transplantation Surgery, Medical University of Warsaw, 02-006 Warsaw, Poland; ^2^Department of Health Psychology, Children's Memorial Health Institute, 00-999 Warsaw, Poland; ^3^CBOS Public Opinion Research Center, 00-503 Warsaw, Poland; ^4^Specialist Hospital in Jaslo, 38-200 Jaslo, Poland; ^5^Polish Transplant Coordinating Center “Poltransplant”, 02-001 Warsaw, Poland; ^6^Department of Surgical and Transplant Nursing, Medical University of Warsaw, 02-007 Warsaw, Poland; ^7^Polish Union for Transplantation Medicine, 00-820 Warsaw, Poland

## Abstract

Public attitude toward deceased donor organ recovery in Poland is quite positive, with only 15% opposing to donation of their own organs, yet actual donation rate is only 16/pmp. Moreover, donation rate varies greatly (from 5 to 28 pmp) in different regions of the country. To identify the barriers of organ donation, we surveyed 587 physicians involved in brain death diagnosis from regions with low (LDR) and high donation rates (HDR). Physicians from LDR were twice more reluctant to start diagnostic procedure when clinical signs of brain death were present (14% versus 5.5% physicians from HDR who would not diagnose death, resp.). Twenty-five percent of LDR physicians (as opposed to 12% of physicians from HDR) would either continue with intensive therapy or confirm brain death and limit to the so-called minimal therapy. Only 32% of LDR physicians would proceed with brain death diagnosis regardless of organ donation, compared to 67% in HDR. When donation was not an option, mechanical ventilation would be continued more often in LDR regions (43% versus 26.7%; *P* < 0.01). In conclusion, low donation activity seems to be mostly due to medical staff attitude.

## 1. Introduction

Transplantation has become a nearly universal therapy of choice for patients with organ failure. The number of patients waiting for solid organs is on the rise in every country. Despite all efforts, the number of available organs is inadequate and 5–25% of all patients on the waiting list will die [1–3]. Organ transplantation is one of the few medical procedures which cannot be carried out without positive attitude, understanding, and cooperation of the whole society.

It has been often believed that low deceased donors (DD) organ donation rate is mainly due to the poor societal perception and nonacceptance (for a number of reasons) of brain death concept. However, several studies have shown that perception of organ donation and transplantation in lay society and their attitude toward transplantation are (at least theoretically) generally positive [4–6]. Factors affecting and improving this attitude have been identified. Wakefield et al. [7] published a review on society's opinions toward organ donation based on 33 most relevant studies regarding this topic. The results confirmed that younger people, especially women, of higher socioeconomic status, educated, with knowledge and awareness of organ donation, who personally knew an organ donor or recipient, with positive family attitude and altruistic beliefs are more willing to donate. People of various medical professions, health care administration, and managers are also a part of the society. A number of studies demonstrated that the attitude of physicians, nurses, and hospital staff toward organ recovery from DD and transplantation is of utmost importance [8–11].

A complex process of donation starts with the identification of the potential donor (i.e., identification of a brain dead patient or patient with irreversible cerebral damage), diagnosis of death, communication with the family of the deceased, and a number of equally important logistic procedures. Hence, in addition to the societal perception and acceptance process, there are three important factors which determine donation rate [12]:

- Physicians' and hospital staff attitude to donation and knowledge of the process of organ donation and transplantation,
- proficiency in recognition of brain death and maintenance of organ function after death,
- type of authorization for organ recovery (opt-in, opt-out), which in some way determine the communication with the family.

**Figure 1:**
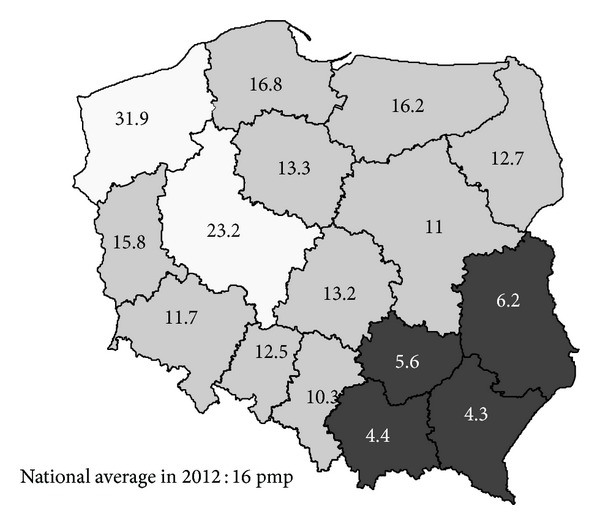
Average organ donations per million population per year across Poland in 2007–2012. HDR regions shown in white and LDR in black.

Deceased donors' organ recovery rate in Poland has never been satisfactory. In 2012 mean donation rate from brain-dead donors achieved European average of 16 pmp [13]. However, the donation rate is not uniform across the country (see Figure 1) and differs substantially from region to region, with two opposite poles of southeastern Poland (5–12 donors pmp) and northwestern parts of the country (20–30 pmp).

In 2012 Public Opinion Research Center CBOS published the results of a survey on public attitudes (1116 respondents) toward organ transplantation across the whole country [14]. The outcomes showed rather positive attitude toward DD organ recovery. Seventy-four percent of respondents would agree to donate their own organs after death and 85% would not object to organ recovery from the family member (provided they knew that deceased person had not objected to donation). Respondents who were against (15%) were older and less educated. Surprisingly enough, religiousness (measured by declared activity in attending a church) did not influence the decision. However, of those not consenting to donation, 23% stated that this would be against their religious believes. Other reasons for objection were interference with corpse integrity (17%), lack of knowledge and understanding of the procedure (14%), emotional reaction (8%), and distrust in medical profession (5%). Nearly half of the population of Poland (49%) believes that death can be only recognized by irreversible arrest of the heart beat and circulation but 44% accepted the concept of the brain death. The report also proved that the general public is not familiar with Transplantation Act [15]. Only 14% of people know that opt-out (“presumed consent”) system is legally binding. Interestingly, in terms of consent to donation and public awareness, the CBOS survey showed no differences between the low and high donation rate regions of the country. Actual donation refusal rate is very low, averaging 9.3% in 2012. Accordingly, this index was similar in LDR (7.4%) and HDR (7.5%) regions of Poland [13].

We presumed that the attitude toward recognition (and acceptance) of brain death (BD) and organ recovery may differ among physicians working in different parts of the country. Hence, a study was designed to examine knowledge and attitude toward brain death and organ transplantation among physicians involved in a care of potential donors in regions of Poland with low and high donation rates.

## 2. Material and Methods

The study was designed by the authors and conducted with help of professional interviewers from Public Opinion Research Center (CBOS) between 11 June and 10 July 2012. Five hundred and eighty-seven anesthesiologists, neurologists, and neurosurgeons (i.e., physicians involved in the process of brain death diagnosis) were interviewed with the PAPI methodology (paper and pen interviewing, a quantitative method of interviewing with a printed questionnaire). All physicians were employees of 57 active donor hospitals or hospitals with potential of donation and represented 50% of aforementioned specialists in their service area. Fifty-five percent of them were women, and average age was 45 years and mean work experience 18.6 years. Fifty-nine percent were anesthesiologists or residents in anesthesiology and intensive care, 35.1% were neurologists or neurology residents, and the rest were neurosurgeons or neurosurgery trainees. The majority were employed in district (48.9%) or provincial (26.7%) hospitals; 10.4% came from university hospitals located in 6 of the 16 provinces of Poland. Four provinces (Lubelskie, Podkarpackie, Swietokrzyskie, and Malopolskie: *n* = 442 interviewed physicians) were of low donation rate (LDR: mean donation rate 4.3–6.2 donations per million population) and the other two were (Wielkopolskie, Zachodniopomorskie: *n* = 145 interviewed physicians) of high donation rate (HDR: mean 23.2 and 31.9 donations pmp in 2007–2012). More detailed characteristics of interviewed physicians are shown in Table 1.

The questionnaire included aspects of diagnosis of brain death and decision making in life supporting therapy and in organ donation process. In addition, respondents were asked to indicate the most important barriers to organ procurement and what, from their perspective, could be done to improve DD organ recovery in their region.

Statistical analysis was made with STATISTICA 10 software (StatSoft Polska, Cracow). Categorical data were assessed with chi-square or Mann-Whitney test; unpaired* t*-test was used for continuous data.

## 3. Results

**Figure 2:**
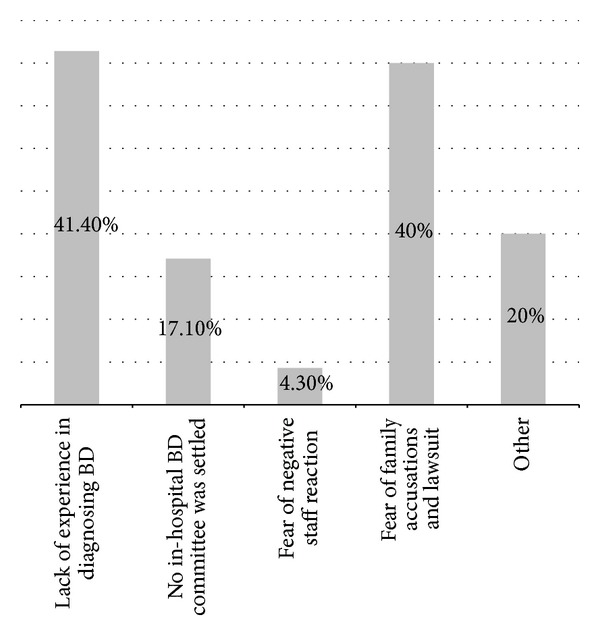
What are the reasons for not proceeding to formal diagnosis when clinical signs of BD are present? (*n* = 70 respondents).

**Figure 3:**
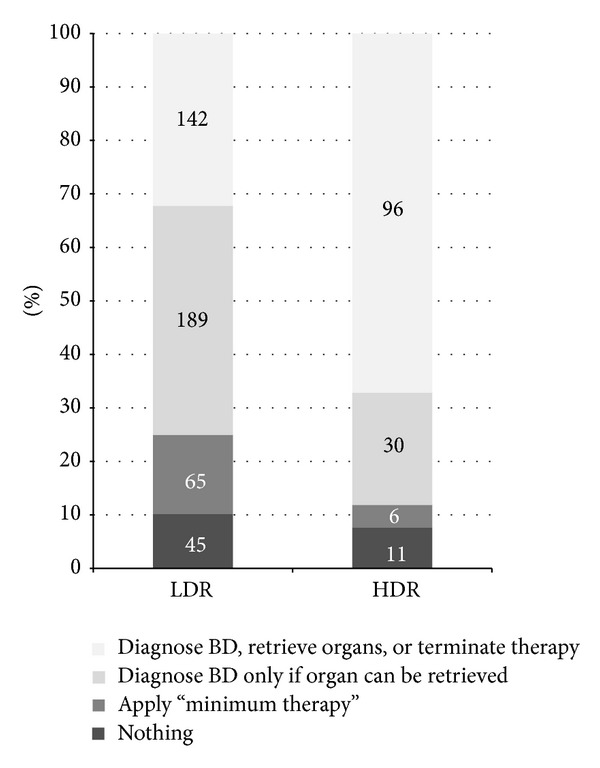
What would you do if clinical signs of brain death were present?

Physicians from the LDR regions were more reluctant to start formal diagnostic procedure when clinical signs suggesting brain death (BD) were present than their colleagues from HDR regions (14.1% versus 5.5% would not initiate the process to diagnose death according to neurological criteria, resp.; *P* < 0.01). Explanations given by 70 respondents reluctant to begin with formal BD diagnosis are summarized in Figure 2. When asked what would they do when clinical signs of brain death were present, 24.9% of physicians from LDR, compared to 11.9% from HDR regions, responded that they would either continue intensive therapy without diagnosing brain death or switch to the so-called minimal therapy (Figure 3). Forty-three percent of respondents from LDR regions (as opposed to 21% from HDR; *P* < 0.001) would diagnose brain death only if organ procurement was considered. Interestingly, residents were in general more willing to diagnose brain death than established specialists (47.4% versus 38.9%, resp., *P* < 0.05) regardless of the region.

Respondents were also asked about further management after formal BD diagnosis, when organ recovery was not an option. Again, 42.8% of physicians from LDR areas would continue with mechanical ventilation until spontaneous circulatory arrest, compared to 26.7% of respondents from HDR regions (*P* < 0.01).

When asked what were the barriers in organ donation, LDR physicians more often claimed that they were due to the poor relation with potential donor's family, deficiency in communication skills, and lack of experience in carrying out the procedure. Lack of confidence in brain death diagnosis also proved to be an important factor (Table 2). As insufficient remuneration was indicated as a reason for not contracting extra duties associated with BD diagnosis, respondents were also asked if they are compensated for care of a potential donor according to the official Ministry of Health Regulation on 19 October 2012. Significantly more physicians from LDR (82.5% versus 63%, *P* < 0.001) complained that they received reduced or no profit at all.

Finally, respondents were asked what measures should be taken in order to increase organ recovery from the deceased donors. They indicated education, experience, need for sharing standards of donor management, training in communication skills, and good cooperation of the ICU and the rest of hospital staff as the most important components of successful donation program.

## 4. Discussion

Not surprisingly, the number of patients awaiting organ transplantation in Poland exceeds the number of available organs; hence, 5% to 10% of potential recipients die each year before receiving the treatment. As of January 1, 2013, over 1300 patients awaited kidney transplantation, 250 patients were enlisted for the heart, and 180 were enlisted for liver transplantation [16]. There were 786 potential deceased donors identified in 2012 across the country, and 615 of them turned out into real donors (at least one organ recovered). Organs were not procured from 73 potential donors due to objection of a family of the donor or of a prosecutor [13]. On the other hand, the number of potential deceased donors in 2012 estimated according to DOPKI (Improving the Knowledge and Practices in Organ Donation) donation index conservatively could have been at least as high as 1450 [17]. This shows that a substantial proportion of patients, in whom brain death could have been diagnosed, die in ICUs not being identified.

Despite declared positive attitude toward organ transplantation, the society as a whole is still not prepared for its full acceptance when the death sets foot in our life. Unconsciousness of legal regulations and unawareness of wishes of family members pertaining organ donation create difficult situation. Despite the presumed consent regulations in Poland, the family of the deceased is always approached to find out what were the wishes of the deceased during his/her lifetime. Such conversation requires proper knowledge, experience, and skills. Refusal rate (objections expressed by the family members) is relatively low (5–15%) and is similar in various parts of the country. Thus, inadequate organ donation activity cannot be explained solely by the barriers within the society.

Our study proved that the official brain death determination procedure is carried out to terminate unnecessary life supporting therapy and consider organ procurement in less than a half (45%) of hospitals. In 36% of hospitals brain death is determined only when organ procurement is planned. In the remaining 19% diagnostic procedure is not undertaken at all and the “therapy” is continued, exploiting resources of the National Health Fund. There was a clear difference between low and high donation activity regions. Moreover, in southeastern parts of the country (LDR), the therapy is continued more often despite formal diagnosis of brain death. Although the Transplantation Act states that every brain death diagnosis should result in termination of therapy with organ recovery or switching off mechanical ventilation, nonadherence is not penalized.

The biggest obstacles indicated by physicians in our study were related to communication with family of the deceased. Psychological situation of a physician, who used to care for a patient trying to save his/her life and was perceived by patient's family as competent and granting some hope is difficult, as in the face of brain death he loses his privileged position. Practitioner has to confront the reaction and emotions of the family after a loss they suffered. Conversation with the family members is difficult and requires knowledge and a high level of interpersonal communication skill and perfection. It may explain physicians' passive attitude in various stages of organ donation: not diagnosing brain death, not switching off the ventilation, and avoiding conversation with deceased' family. Similar reluctance to act and communicate with family was observed by Exley et al. who surveyed 1,650 Texas physicians and found that they were reluctant to approach grieving families and would only do it when positive response was expected. Of a total 28% of practitioners who had experienced a situation when their patient was diagnosed with BD, only 40% had approached the family, 19% ensured someone else did the job, and 17% took no action whatsoever [15].

Respondents of the survey expressed the need for standards and training in organ donation. To learn how to manage severe stress effectively, apart from instrumental methods, one has to develop psychological skills in communication and dealing with emotions. Few physicians are naturally flexible, easily adjust to an interlocutor, and are able to present adequate arguments. Most of them need education in humanities and learning psychological skills of communicating in difficult situations. Bøgh et al. show a need for training not only in medical issues (potential donor identification) but also in psychological ones (information and support to a deceased relatives) as well [18]. Psychosocial skills not only improve professional competence of a physician but also ameliorate atmosphere in a place of work, which was also ranked high in our study in terms of increasing the numbers of procured organs. Optimal team communication and interrelations increase sensation of safety and professional satisfaction, which seems of utmost importance in an ICU environment.

Additional important factor which should be taken into account is opinion of the physician regarding brain death. It is quite possible that some of the professionals are not fully convinced that recognition of brain death equals the death of a person and hence, educational actions are needed.

Another hypothesis explaining limitations within medical society pertains authority, societal position of physicians, and public trust in this group [7]. In Poland they are surprisingly low. According to Wakefield and previous Polish studies [19], factors affecting organ donation are older age, distrust, fear of organ misuse. The family (parents) of potential donors is usually not too young, which shapes their relation with a physician and attitude to donation. Low societal trust and anxiety of negative perception can drive a practitioner to try to improve his image and receive societal acceptation of his actions. It may result in a trend to publicly adhere strictly to rigid moral norms, distance from complicated ethical and moral issues, and avoid difficult tasks. In general, study results show that barriers to increase the number of organs available for transplantation are found equally on the side of “lay” (in terms of medicine) society and the professionals. The results suggest the need for mutual understanding and overtness. Prospect of donation ought to bring people together and not to antagonize physicians with the society.

An Australian study among intensivists (*n* = 285) showed almost universal (99%) support for organ donation, with 89% of physicians being registered as donors and 94% who said they would support donation from a dependent [20]. However, 73% admitted that requesting organ donation from patients' families was both stressful and unpleasant. Family distress was one of the main reasons not to ask for donation. Molzahn found it difficult for 47% of Canadian ICU physicians to explain brain death concept to families [21] and for as many as 85% of nurses to approach families for donation [22]. According to physicians in our study, lack of experience in communication with donor family, unfamiliarity with donor identification, and brain death diagnosis procedures all ranked very high as obstacles in donation process. This strongly indicates knowledge deficiencies (68% of physicians and 71% of nurses answered correctly to questions testing their knowledge of brain death and organ donation) or inadequate training in stressful experience such as discussing the problem with the grieving relatives. These factors, coupled with concerns and fears about the procurement process, may be affecting actual involvement in organ procurement practice. Although health care practitioners exhibited strong approval, they lacked understanding of key facts, and educational programs as well as an in-house coordinator are needed to increase awareness of organ donation and transplantation in nontransplant hospitals. Knowledge of donation process was recognized by many studies reviewed by Walters to be influential on general attitudes, with 58% of papers recommending education and training [23]. Educational shortage may easily lead to results observed by Abbud-Filho et al, who found that 15% of their doctors refused to acknowledge that BD patients were potential organ donors [24]. This could partially explain the difference in donation rate not only among two studied regions of Poland but also among various European countries. Some surprising evidence was found by Gaber et al. [25] and Pugliese et al. [26]. 35% and 7% of ICU personnel and physicians, respectively, did not believe brain death was equivalent to a death of an individual. How could one then expect them to approach a grieving relative of a BD patient and request organ donation?

Our study shows that the donation rate may be primarily affected by physicians' and all hospital personnel attitudes toward brain death recognition and organ donation. Knowledge and proficiency in recognition of brain death and the support of organ function after death are of utmost importance. However, donation activity in the nontransplant hospitals requires proper atmosphere, positive approach toward the process of all hospital health care professionals and administration, and excellently trained coordinator as a team leader.

## Figures and Tables

**Table 1:** Physicians who participated in the survey.

	LDR *n* = 442	HDR *n* = 145	*P*
M/F	182/259	83/62	0.001

Age (yrs)	44.3 ± 10.7	46.1 ± 11.6	0.09

Professional experience (yrs)	18.1 ± 10.8	20 ± 11.9	0.1

Anesthesiologists	198 (44.8%)	71 (49%)	0.57
Anest. residents	56 (12.7%)	19 (13.1%)	
Neurologists	123 (27.8%)	30 (20.7%)	
Neurl. residents	42 (9.5%)	12 (8.3%)	
Neurosurgery	18 (4.1%)	11 (7.6%)	
Nueros. residents	5 (1.1%)	2 (1.4%)	

Practice hospital			
University	47 (10.6%)	14 (9.6%)	
Provincial	97 (21.9%)	60 (41.4%)	0.01
District	235 (53.2%)	52 (35.9%)	
Municipal	63 (14.2%)	19 (13.1%)	

**Table 2:** Obstacles in identification of potential deceased donor according to 587 interviewed physicians*.

Factor	LDR	HDR	*P*
Poor relations with a family of deceased patient	5.2 ± 3.4	4.5 ± 3.1	0.04
Lack of experience in communication with DD family	4.8 ± 3.2	4.1 ± 3.2	0.04
Unfamiliarity with the procedure of potential donor identification	4.4 ± 3.4	3.1 ± 3.3	0.001
Professional burnout	3.7 ± 3.3	3 ± 3.2	0.03
Diffidence in brain death diagnosis procedure	3.5 ± 3.2	2.6 ± 3	0.004
Whole team indifference to the demand of organs for transplant	3.2 ± 3.1	2.6 ± 3.1	0.05
Concern about suspicion of abuse or exceeding one's competence	3.3 ± 3.5	2.5 ± 3.3	0.02
Low fiscal motivation	2.8 ± 3.5	2.1 ± 3.1	0.03
Conflicts within the team	1.5 ± 2.4	1.3 ± 2.3	0.5
Open or covert reluctance of the superiors	1.4 ± 2.4	1.2 ± 2.4	0.6

*Physicians were asked to assign the number of points from 0 to 10 to each factor, with 0 meaning totally insignificant and 10 a factor of crucial importance.

## References

[1] Valapour M, Paulson K, Smith JM (2013). OPTN/SRTR 2011 annual data report: lung. *The American Journal of Transplantation*.

[2] Colvin-Adams M, Smith JM, Heubner BM (2013). OPTN/SRTR 2011 annual data report: heart. *The American Journal of Transplantation*.

[3] Kim WR, Stock PG, Smith JM (2013). OPTN/SRTR 2011 annual data report: liver. *American Journal of Transplantation*.

[4] Rey JW, Grass V, Barreiros AP (2012). Organ procurement in Germany a regional survey among students. *Deutsche Medizinische Wochenschrift*.

[5] Frutos MA, Blanca MJ, Ruiz P, Mansilla JJ, Seller G (2005). Multifactorial snowball effect in the reduction of refusals for organ procurement. *Transplantation Proceedings*.

[6] Cucchetti A, Zanello M, Bigonzi E (2012). Te use of social networking to explore knowledge and attitudes toward organ donation in Italy. *Minerva Anestesiologica*.

[7] Wakefield CE, Watts KJ, Homewood J, Meiser B, Siminoff LA (2010). Attitudes toward organ donation and donor behavior: a review of the international literature. *Progress in Transplantation*.

[8] Abidin ZLZ, Ming WT, Loch A, Hilmi I, Hautmann O (2013). Are health professionals responsible for the shortage of organs from deceased donors in Malaysia?. *Transplant International*.

[9] Smudla A, Mihály S, Ökrös I, Hegedus K, Fazakas J (2012). The attitude and knowledge of intensive care physicians and nurses regarding organ donation in Hungary—it needs to be changed. *Annals of Transplantation*.

[10] Rios A, Conesa C, Ramirez P (2006). Attitudes of resident doctors toward different types of organ donation in a Spanish transplant hospital. *Transplantation Proceedings*.

[11] Chernenko SM, Jensen L, Newburn-Cook C, Bigam DL (2005). Organ donation and transplantation: a survey of critical care health professionals in nontransplant hospitals. *Progress in Transplantation*.

[12] Flodén A, Kelvered M, Frid I, Backman L (2006). Causes why organ donation was not carried out despite the deceased being positive to donation. *Transplantation Proceedings*.

[13] Antoszkiewicz K, Parulski A, Trujnara M, Czerwinski J (2013). Pobieranie i przeszczepianie narzadow w Polsce w 2012 r. *Poltransplant Biuletyn Informacyjny*.

[14] Feliksiak M Attitude to organ transplantation. http://cbos.pl/SPISKOM.POL/2012/K_105_12.PDF.

[15] Exley MH, Serbin MF, Goldstein RM (1992). Approaching families for organ donation: physicians are willing. *Texas Medicine*.

[16] Lewandowska D, Hermanowicz M, Przygoda J, Marcinkowska J, Podobińska I (2013). Krajowa Lista Oczekujących na Przeszczepienie. *Poltransplant Biuletyn Informacyjny*.

[17] Amil M, Brezovsky P, Czerwinski J, Coll E, Collet D, de Guerra A (2009). *Guide of Recommendations for Quality Assurance Programmes in the Deceased Donation Process*.

[18] Bøgh L, Madsen M (2005). Attitudes, knowledge, and proficiency in relation to organ donation: a questionnaire-based analysis in donor hospitals in northern Denmark. *Transplantation Proceedings*.

[19] Jakubowska-Winecka A, Rowiński W, Włodarczyk Z, Wójtowicz S (2006). Extreme attitudes toward organ transplantation: how do supporters and opponents of this method of treatment differ in Poland?. *Transplantation Proceedings*.

[20] Pearson IY, Zurynski Y (1995). A survey of personal and professional attitudes of intensivists to organ donation and transplantation. *Anaesthesia and Intensive Care*.

[21] Molzahn AE (1997). Knowledge and attitudes of physicians regarding organ donation. *Annals of the Royal College of Physicians and Surgeons of Canada*.

[22] Molzahn AE (1997). Knowledge and attitudes of critical care nurses regarding organ donation. *Canadian Journal of Cardiovascular Nursing*.

[23] Walters TP (2009). Are front line health professionals responsible for the organ crisis?. *Journal of Intensive Care Society*.

[24] Abbud-Filho M, Miyasaki MCOS, Ramalho HJ, Domingos N, Garcia R, Pucci F (1997). Survey of concepts and attitudes among healthcare professionals toward organ donation and transplantation. *Transplantation Proceedings*.

[25] Gaber AO, Hall G, Phillips DC, Tolley EA, Britt LG (1990). Survey of attitudes of health care professionals toward organ donation. *Transplantation Proceedings*.

[26] Pugliese MR, Degli Esposti D, Venturoli N (2001). Hospital attitude survey on organ donation in the Emilia-Romagna region, Italy. *Transplant International*.

